# GraphyloVar: predicting the impact of non-coding variants using a multi-species sequence model

**DOI:** 10.1093/bioinformatics/btag426

**Published:** 2026-06-22

**Authors:** Dongjoon Lim, Mathieu Blanchette

**Affiliations:** School of Computer Science, McGill University, Montreal, QC H3A 2A7, Canada; School of Computer Science, McGill University, Montreal, QC H3A 2A7, Canada

## Abstract

**Motivation:**

Understanding the functional impact of genetic variants is a key problem for precision medicine. Tools like CADD, PhyloP, and PhastCons are useful, but they often look at each position in the genome in isolation. This means they can miss important information from the evolutionary history that connects different species. In this paper, we extend our previous model, Graphylo, to predict the effects of variants. Our new model, GraphyloVar, is built to directly utilize the phylogenetic tree that relates the species.

**Results:**

GraphyloVar is a deep learning model that considers both DNA sequence and evolutionary patterns from many species. It uses two main components: Graph Convolutional Networks (GCNs) to process the phylogenetic tree, and Transformer encoders to extract features from the DNA sequences. Pre-trained to predict population-level allele frequencies on the TOPMed whole-genome sequencing cohort, GraphyloVar achieves an AUROC of 0.6246 zero-shot on ∼149M held-out variants, and an ensemble with CADD reaches 0.6442 (+0.020, $P<10^{-15}$). Fine-tuned GraphyloVar achieves the highest AUROC across all 13 MPRA benchmark datasets. By integrating deep learning with explicit phylogenetic input, GraphyloVar offers a powerful and complementary approach to variant effect prediction that utilizes the full evolutionary history from many species to better identify and prioritize important non-coding variants.

**Availability and implementation:**

Code and datasets are available at https://github.com/DongjoonLim/GraphyloVar under DOI: 10.5281/zenodo.20616818.

## 1 Introduction

Understanding the functional consequences of genetic variants is central to precision medicine and the decoding of complex genotype-phenotype relationships. Subtle alterations in the genome can have significant effects on phenotypic outcomes. Large-scale efforts, such as the 1000 Genomes Project and the ENCODE Project (Siva 2008, ENCODE Project Consortium 2012) have shown that the human genome is highly complex, with various regulatory components and many genetic variants whose functions are still not well understood. Although genome-wide association studies (GWAS) have identified numerous genetic variants associated with disease phenotypes, they often do not explain the precise molecular mechanisms through which specific variants exert their effects (Uffelmann *et al.* 2021). Approximately 90% of disease-associated genetic variants are located in non-coding regions, with a likely impact on transcriptional or post-transcriptional regulation. However, the mechanisms through which these non-coding variants influence gene expression, regulatory networks, and downstream cellular processes remain poorly understood.

To address this problem, researchers have turned to computational methods. One common strategy is to use measures of evolutionary conservation. Tools such as PhyloP and PhastCons were developed to quantify the degree of evolutionary conservation at individual genomic positions (Siepel *et al.* 2005, Pollard *et al.* 2010), based on the premise that variants at highly conserved positions are more likely to be functionally deleterious. Following this logic, integrative tools, such as CADD, were created to predict the functional impact of variants by combining these conservation scores with many other types of data, such as functional annotations (Kircher *et al.* 2014).

PHACT (Kuru *et al.* 2022) and its gradient-boosted extension PHACTboost use protein-level features including amino acid conservation, protein structure, and functional domain annotations. These tools are designed specifically for missense variants in protein-coding regions and require protein sequence and structural context as input. GraphyloVar operates in a fundamentally different scope. It predicts the functional impact of both coding and non-coding variants using only the multi-species whole-genome alignment, without any protein-level features. More than 98% of the human genome is non-coding, and the majority of disease-associated variants identified by GWAS fall in non-coding regions where PHACT and PHACTboost cannot be applied. The two approaches are therefore complementary rather than competing.

More recently, a different class of deep learning models, such as DeepSEA and Enformer, have been developed (Zhou and Troyanskaya 2015, Avsec *et al.* 2021). They use long stretches of DNA sequence (typically human) to predict features like chromatin accessibility, histone marks, and gene expression. These models have proven useful for capturing regulatory signals from the sequence. However, both approaches have limitations. The conservation-based methods use evolutionary context but often ignore local sequence patterns. On the other hand, the single-species deep learning models are powerful at using local sequence context but do not explicitly incorporate the broader evolutionary information that comes from comparing multiple species. This limitation is especially pronounced for non-coding regions, where detecting subtle evolutionary constraints requires both local sequence context and multi-species evolutionary signals.

Building on the above contrast between single-species sequence models and approaches that use evolutionary information, other researchers have proposed foundation models for DNA, such as DNABERT (Ji *et al.* 2021). These models are usually trained on sequences from a single species (most often human) and therefore do not leverage cross-species signals. Some recent methods, such as GPN-MSA (Benegas *et al.* 2025), do include multiple sequence alignments (MSAs). However, GPN-MSA treats each alignment column as a single input token, flattening the evolutionary information and discarding the hierarchical phylogenetic structure that relates the species within each column.

In contrast to the approaches discussed above, our method, GraphyloVar, explicitly learns cross-species dependencies with a graph-based architecture. By incorporating MSAs together with their phylogenetic structure from the start of pre-training, it can learn evolutionary constraints across species while preserving the hierarchical relationships between organisms. This design not only improves predictive accuracy, but also maintains biological relevance by preserving the evolutionary context that many other methods overlook. Building on our previous work with Evolstm (Lim and Blanchette 2020), where we showed that sequence context strongly influences mutation probabilities, and Graphylo (Lim *et al.* 2024), where we showed that incorporating evolutionary information improves the prediction of regulatory sites, we extend these ideas to capture both local sequence context and cross-species evolutionary patterns through explicit phylogenetic modeling.

A key difference between GraphyloVar and GPN-MSA is the explicit modeling of phylogenetic topology. Whereas GPN-MSA flattens alignment columns into single tokens, GraphyloVar passes per-species features through a Graph Convolutional Network (GCN) whose adjacency matrix encodes the mammalian phylogenetic tree, preserving the hierarchical evolutionary relationships among species.

A distinctive feature of the GraphyloVar framework is its pre-training objective: rather than predicting masked nucleotides, the model predicts population-level allele frequencies. This biologically informed objective yields a foundation model that captures the evolutionary constraints shaping genomic variation, applicable as a zero-shot predictor or fine-tuned for specific downstream tasks.

## 2 Materials and methods

GraphyloVar is a deep learning model developed to predict the functional impact of non-coding variants by modeling their full sequence and evolutionary context. The model takes as input a 65 bp human DNA sequence centered on the variant of interest, together with orthologous sequences from 57 other mammals and 57 computationally reconstructed ancestral sequences (Blanchette *et al.* 2004b, Miller *et al.* 2007). All sequences are organized by a known phylogenetic tree, so that each observed or reconstructed sequence is associated with a tree node, and their relationships follow the tree topology.

The GraphyloVar architecture is hybrid. Transformer encoders extract context-dependent sequence features within the 65 bp input window (Vaswani *et al.* 2017). Graph Convolutional Networks (GCNs) propagate information along the phylogenetic tree, allowing the model to learn how the nucleotide at a position in one species informs the probabilities of nucleotides at the same position in related species (Kipf and Welling 2016). By combining sequence features with the explicit tree structure, GraphyloVar uses both local sequence context and cross-species information, enabling more accurate predictions than models that use single-sequence inputs or simple conservation scores.

### 2.1 Data preprocessing

We utilized MSAs from the 100-way whole-genome alignment of vertebrates provided by the UCSC Genome Browser (Blanchette *et al.* 2004b, Miller *et al.* 2007). For our analysis, we extracted sequences for only the 58 extant placental mammalian species. We restricted the analysis to placental mammals because the Ancestors1.0 ancestral reconstruction achieves higher accuracy within this clade, where the phylogeny is well-established and branch lengths are well-calibrated (Blanchette *et al.* 2004a); non-mammalian vertebrate sequences were therefore excluded.

To reconstruct the evolutionary history of these sequences, we inferred ancestral genome sequences at the internal nodes of the mammalian phylogenetic tree using the Ancestors1.0 program (Blanchette *et al.* 2004a, Diallo *et al.* 2010). This maximum-likelihood approach reconstructs the most probable nucleotide at each position for ancestral species based on the sequences of their descendants and the known phylogenetic relationships. The process resulted in a dataset comprising 115 sequences per alignment block: 58 from extant species and 57 from inferred ancestors. For model input, we segmented the genome-wide alignment into 65 bp blocks centered on each variant position, a window size that captures sufficient local sequence context while remaining computationally tractable.

### 2.2 Model architecture

**Figure 1:**
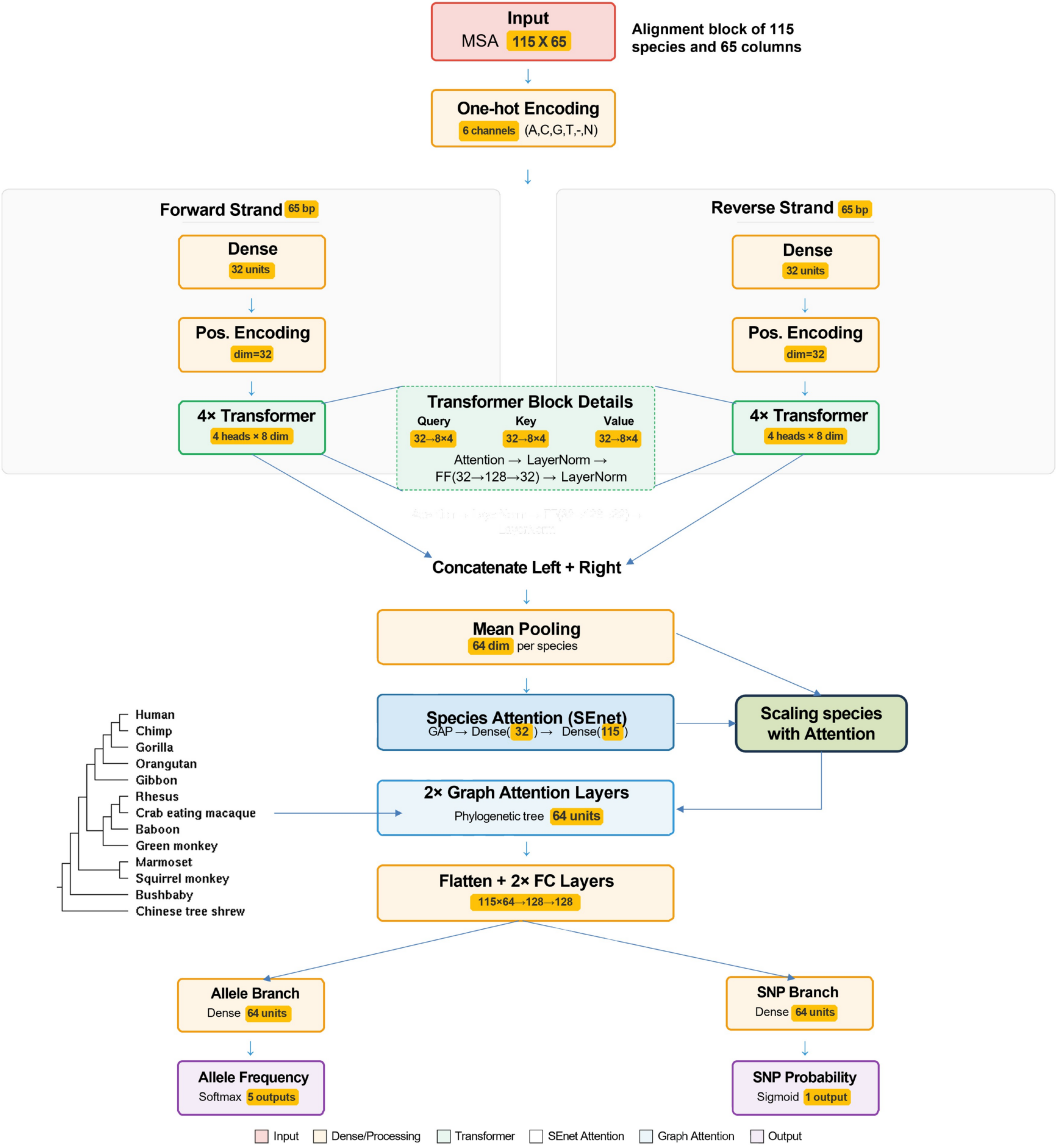
Overview of GraphyloVar’s architecture. For a given 65 bp human sequence and its orthologous and inferred ancestral sequences across 115 placental mammals (58 extant species + 57 ancestral nodes), each species’ sequence is one-hot encoded and split into forward and reverse strands. Each strand passes through an initial dense layer (32 units) with batch normalization and GELU activation, followed by sinusoidal positional encoding. Two Transformer encoder layers process each strand independently (four attention layers total, forward and reverse complement), capturing sequence patterns and dependencies across the 65 bp window. The output for each strand is mean-pooled over the 65 positions to produce a 32-dimensional representation, and the two strand representations are concatenated to form a 64-dimensional feature vector per species. A Squeeze-and-Excitation block produces per-species attention weights before these features feed into a two-layer Graph Convolutional Network (GCN) that propagates information along the phylogenetic tree. The GCN output is flattened and passed through a shared fully connected layer (128 units, ReLU) before branching into dual prediction heads: one for allele frequency prediction (a 64-unit dense layer with softmax over A, C, G, T, gap) and another for SNP probability prediction (a 64-unit dense layer with sigmoid). This architecture enables GraphyloVar to simultaneously model nucleotide preferences and variation tolerance at each genomic position.

The GraphyloVar architecture consists of two main parts that work in sequence, as shown in Fig. 1. First, the Transformer blocks learn features from aligned DNA sequences (Vaswani *et al.* 2017). Then, the output of the Transformers is passed to a Graph Convolutional Network (GCN), which processes these features using the structure of the phylogenetic tree (Kipf and Welling 2016). These two modules work together to integrate sequence information with broader evolutionary signals.

#### 2.2.1 Embedding and transformer blocks

For each species, sequences are one-hot encoded using a six-letter alphabet (A, C, G, T, -, N). Each 65 bp sequence is then paired with its reverse complement, and both strands are processed in parallel through the same Transformer pathway. The one-hot encoded nucleotides first pass through a dense layer with 32 units, followed by batch normalization (Ioffe and Szegedy 2015), a GELU activation function (Hendrycks and Gimpel 2016), and dropout (Srivastava *et al.* 2014), projecting each one-hot vector into a 32-dimensional embedding space. We then add a sinusoidal positional encoding, enabling subsequent transformer layers to capture position-dependent sequence patterns.

For both the forward and reverse strands, the model applies two Transformer blocks in series per strand (four total, processing forward and reverse complement independently) (Vaswani *et al.* 2017). Transformers are suitable for genomic sequence analysis (Ji *et al.* 2021, Benegas *et al.* 2025). Their main advantage is self-attention, which enables the model to attend to different sequence patterns simultaneously, creating context-aware representations that capture long-range dependencies across the input window. After transformer processing, the output sequence for each strand is mean-pooled over the 65 input positions, producing a 32-dimensional representation per strand. The forward and reverse complement strand representations are then concatenated into a 64-dimensional feature vector per species. This concatenated vector is passed to the SE block and GCN.

#### 2.2.2 Species attention and graph convolution

We employ a species attention mechanism, which is a form of Squeeze-and-Excitation (SE) block (Hu *et al.* 2018). The SE block applies global average pooling to each species’ feature vector, passes the result through two fully connected layers with a sigmoid activation to produce species-wise attention weights, and multiplies these weights back onto the original feature representations before the GCN layer.

To model evolutionary relationships, GraphyloVar uses a two-layer GCN on the phylogenetic tree (Kipf and Welling 2016). The nodes correspond to extant or ancestral species. Edges indicate evolutionary relationships. The GCN layers propagate information between connected nodes in the tree, so each species’ representation is updated using information from its direct relatives (parent and child nodes). This enables the model to capture functional elements conserved among related species while retaining sensitivity to species-specific patterns. The layer update is:


$$H^{\left(l+1\right)}=\sigma \left({\tilde{D}}^{-\frac{1}{2}}\tilde{A}{\tilde{D}}^{-\frac{1}{2}}H^{\left(l\right)}W^{\left(l\right)}\right)$$

Here, $H^{\left(l\right)}$ is the node-feature matrix at layer *l*. The matrix $\tilde{A}=A+I$ is the adjacency with self-loops, and $\tilde{D}$ is its degree matrix. The matrix $W^{\left(l\right)}$ is learnable. The function σ is the GELU nonlinearity (Hendrycks and Gimpel 2016). The second layer outputs a 32-dimensional embedding for each species.

#### 2.2.3 Dual-output prediction heads

After the GCN, all species embeddings are flattened and passed through a shared fully connected layer (128 units, ReLU activation, dropout). The shared representation then branches into two prediction heads:

- Allele frequency prediction. A dense layer with 64 units (ReLU, dropout), followed by a final dense layer with softmax outputs a distribution over five states: A, C, G, T, and a gap/unknown state.
- SNP probability prediction. A parallel dense layer (64 units, ReLU, dropout), followed by a final sigmoid neuron predicting the probability that a position is a SNP.

This dual-output setup provides complementary signals. The allele head models nucleotide preferences. The SNP head models tolerance to variation. Together, they support downstream assessment of variant effects in a consistent framework. A model component ablation confirms that each architectural element contributes incrementally: the Transformer-only baseline, TF + GCN tree, and full model (with the species-attention SE gate) each yield progressively higher held-out AUROC across all genomic region categories (Fig. S1, available as supplementary data at *Bioinformatics* online).

### 2.3 Model training and optimization

GraphyloVar uses a biologically informed pretraining task. We mask only the central human nucleotide in each MSA block. This masking strategy targets the position most directly relevant to variant effect prediction. Unlike masked language models that predict the most probable nucleotide at masked positions (Ji *et al.* 2021, Benegas *et al.* 2025), the model predicts the full population-level allele frequency distribution at the masked locus. The training labels come from the Trans-Omics for Precision Medicine (TOPMed) dataset (Burgess 2021), which is a large NIH program with whole-genome sequencing across multiple ancestries. We pre-train the model with the Adam optimizer (Kingma and Ba 2015) using the default settings in TensorFlow 2.5 (Abadi *et al.* 2015). Mini-batch size is 64 for the main model (flank =32). For the allele frequency head, we use categorical cross-entropy loss, which is suitable for predicting a probability distribution over five allele states (A, C, G, T, gap). For the SNP probability head, we use binary cross-entropy loss. GELU activation is used in the Transformer encoder and GCN layers; the prediction heads use ReLU. To limit overfitting, we apply dropout with rate 0.2 and early stopping based on validation loss. The main model is trained on TOPMed variants from chromosomes 1–10, validated on chromosomes 11–12, and evaluated on the held-out test set comprising chromosomes 13–22 (∼149M variants).

After the pre-training phase, the model can be used in two primary ways. First, it can be applied directly as a foundation model for zero-shot prediction on various tasks. Alternatively, it can be fine-tuned on specific downstream tasks if labeled data is available. For task-specific fine-tuning, we updated all model weights rather than freezing the pre-trained layers.

We tuned the key hyperparameters of the model by evaluating a few candidate values for each parameter while keeping the others constant. The configuration we found was 2 Transformer layers per strand (4 total), 2 GCN layers with 32-dimensional node embeddings, and a shared fully connected layer with 128 units. This configuration achieved strong predictive performance while remaining computationally tractable.

### 2.4 GraphyloVar score: quantifying variant impact

GraphyloVar’s dual-output architecture provides two key pieces of information at each genomic position: (i) a probability distribution over the five allele states {A,C,G,T,gap}, and (ii) the probability that the position is polymorphic (i.e. a SNP). To create a measure of a variant’s predicted impact, we combine these two outputs into a single score using the reference and alternate nucleotide probabilities.

For a variant at position *i*, let $p_{a}=P({\text{alt}}_{i}|{\text{MSA}}_{i},\Theta )$ and $p_{r}=P({\text{ref}}_{i}|{\text{MSA}}_{i},\Theta )$ denote the model’s predicted probabilities for the alternate and reference alleles, and let $p_{s}=P({\text{SNP}}_{i})$ denote the predicted probability that position *i* is polymorphic.

The first component is the *Allele Probability Ratio*, a log-ratio of the alternate- and reference-allele probabilities:


(1) $${\text{Allele Ratio}}_{i}=\text{log}\;\left(\frac{p_{a}}{p_{r}}\right).$$

A lower, more negative score suggests that the alternate allele is much less probable than the reference allele. The model captures this through learned sequence and evolutionary patterns that indicate the reference allele is under functional constraint. Therefore, a substitution to a low-probability alternate allele is likely to be deleterious.

However, the allele ratio alone does not account for overall tolerance for variation. A site might be highly conserved, meaning any change is likely to be damaging, regardless of the specific alternate allele. To address this, the final *GraphyloVar Score* incorporates the predicted SNP probability $p_{s}$ and is defined as:


(2) $${\text{Graphylo Var Score}}_{i}=\text{log}\;\left(\frac{p_{s}p_{a}}{p_{s}p_{r}+(1-p_{s})}\right).$$

This combined score offers a more complete picture because if a site is predicted to be highly conserved ($p_{s}$ is low), the numerator becomes small, resulting in a very low (more deleterious) score for any alternate allele. This correctly reflects that changes at invariant sites are likely harmful. It also retains allele-specific constraint: if a site is predicted to be variable ($p_{s}$ is high), the score is mainly driven by the ratio $p_{a}/p_{r}$. Lower GraphyloVar scores indicate variants predicted to be more damaging.

### 2.5 Baseline approaches and datasets

To evaluate GraphyloVar’s performance, we conducted comparisons against diverse established methods for variant effect prediction. These baseline methods represent a spectrum of approaches, from traditional conservation metrics to advanced machine learning models, each capturing different aspects of genomic function.

#### 2.5.1 PhyloP

PhyloP measures evolutionary constraint at individual nucleotides by comparing observed substitution rates to neutral evolution (Pollard *et al.* 2010). It produces site-specific scores where positive values indicate conservation. Although effective for single-site constraint scoring, it does not capture broader sequence context. We utilized 100-way vertebrate PhyloP scores from the UCSC Genome Browser.

#### 2.5.2 PhastCons

In contrast to PhyloP, PhastCons uses a hidden Markov model to identify contiguous conserved elements (Siepel *et al.* 2005). It models phylogenetic relationships to detect genomic stretches under purifying selection, making it suitable for regulatory elements that function as cohesive units. We used the 100-way vertebrate PhastCons conservation probabilities.

#### 2.5.3 CADD

CADD integrates genomic annotations into a pathogenicity score using a support vector machine trained to distinguish simulated from observed variants (Kircher *et al.* 2014). It combines conservation metrics and functional data to assess potential deleteriousness in both coding and non-coding regions. We used CADD v1.7 scores (GRCh38) for our evaluation.

#### 2.5.4 Enformer

Uses a transformer architecture with self-attention to capture long-range interactions (input window ≈200 kb) (Avsec *et al.* 2021). As the original method does not provide a standard variant effect score, we derived Enformer-based scores in two settings. For the zero-shot MAF discrimination task (Section 3.1), we trained a two-layer fully connected neural network (256 hidden units per layer, ReLU activations, dropout rate 0.3) taking the difference between Enformer’s reference and alternative allele predictions as input features to predict CADD targets, using the same chromosome-level train/val/test split as the main model. For the MPRA benchmarks (Section 3.3), we trained a separate two-layer DNN (same architecture) on Enformer features with MPRA binary labels, using the same leave-one-out cross-validation as the other MPRA models.

#### 2.5.5 GPN-MSA

GPN-MSA is conceptually related to GraphyloVar in that it also uses MSAs as input (Benegas *et al.* 2025). Architecturally, however, it processes concatenated alignment columns through transformers without explicitly modeling the phylogenetic structure among species.

### 2.6 GPN-Star

GPN-Star (Benegas *et al.* 2025) is an MSA-aware extension of GPN-MSA and is included here as a contemporary MSA-based baseline. The large-scale genomic foundation model Evo2 (Brixi *et al.* 2026) requires CUDA compute capability 8.9 or higher (Ada or Hopper class GPUs), which exceeds our current hardware; we therefore exclude it from the quantitative benchmark and leave it as an important future comparison.

These baselines were selected to represent methodologically distinct approaches: conservation metrics (PhyloP, PhastCons), integrative scoring (CADD), sequence-based deep learning (Enformer), and MSA-aware models (GPN-MSA, GPN-Star). Although databases such as dbNSFP aggregate prediction scores from more than 36 tools, many of these share underlying conservation metrics or functional annotation features. We focused on these representative families to cover the major methodological landscape of variant effect prediction while avoiding redundancy in the benchmark.

To evaluate predictions against experimental data, we compiled a broad collection of Massively Parallel Reporter Assay (MPRA) datasets, covering various regulatory contexts and disease associations. For MPRA studies that focus on enhancer and promoter regions, we incorporated data from studies investigating psychiatric disorders, including schizophrenia (Myint *et al.* 2020) and major depressive disorder (Mulvey and Dougherty 2021), as well as neurodegenerative conditions such as Alzheimer’s disease and dementia (Myint *et al.* 2020, Cooper *et al.* 2022). We also included cancer-related datasets that focus on melanoma (Choi *et al.* 2020, Long *et al.* 2022) and multiple myeloma (Ajore *et al.* 2022), along with autoimmune conditions such as lupus (Lu *et al.* 2021). To represent expression-modulating variants in non-disease contexts, we included data from an eQTL-focused reporter assay (Tewhey *et al.* 2016). We also analyzed MPRA studies that focused on 3’ UTR variants. (Griesemer *et al.* 2021) performed a genome-wide functional screen to identify causal 3’ UTR variants relevant to human disease and evolution, using annotations from the GWAS catalog. (Schuster *et al.* 2023) characterized 3’ UTR variants associated with prostate cancer susceptibility. These two datasets form the 3’ UTR MPRA group in Fig. 3. For genome-wide MPRA analyses spanning diverse loci and regulatory contexts, we incorporated three additional datasets. (Abell *et al.* 2022) measured the regulatory effects of variants at multiple fine-mapped GWAS loci to identify causal alleles underlying genetic associations in humans. McAfee *et al.* (2023) systematically investigated the allelic regulatory activity of schizophrenia-associated common variants across 5,173 fine-mapped GWAS loci. These three datasets form the genome-wide analyses group in Fig. 3.

### 2.7 Implementation

GraphyloVar was implemented in Python 3.9 using TensorFlow 2.5 on NVIDIA Quadro RTX 6000 GPUs (compute capability 7.5). Code is available at https://github.com/DongjoonLim/GraphyloVar.

## 3 Results

### 3.1 Differentiating common variants from rare variants

To evaluate GraphyloVar’s ability to capture evolutionary constraints that influence allele frequencies, we assessed its zero-shot performance (using the model directly after pre-training without task-specific fine-tuning). Specifically, we tested whether the model could distinguish between common variants (Minor Allele Frequency (MAF) ≥ 0.01) and rare variants (MAF < 0.001) in the human population. We used a chromosome-level holdout strategy: chromosomes 13–22 were excluded entirely from pre-training and validation. All evaluation variants were drawn exclusively from these held-out chromosomes, yielding approximately 149 million held-out SNVs from the TOPMed whole-genome sequencing cohort (Burgess 2021). This chromosome-level partition eliminates positional leakage while ensuring evaluation variants are drawn from a diverse genomic background. This distinction is biologically important, as variants under stronger purifying selection tend to be maintained at lower frequencies in the population.

**Figure 2:**
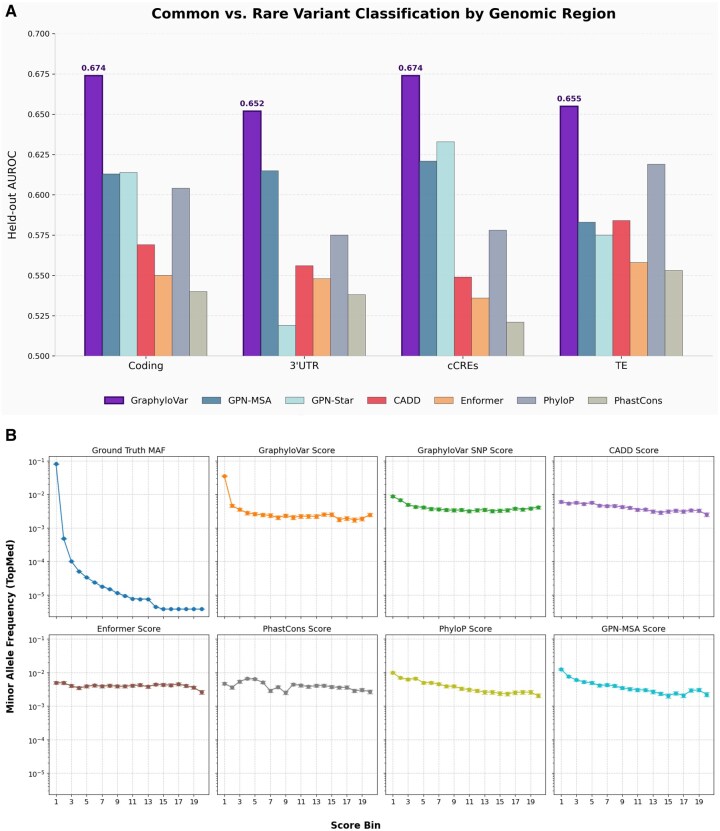
GraphyloVar’s performance distinguishing common from rare variants. (A) AUROC for four UCSC-annotated genomic region types (Coding, 3’ UTR, cCREs, TE), evaluated on held-out variants from chromosomes 13 to 22. Only variants with a region annotation are included; unannotated intergenic variants are excluded from this panel (see Fig. S1, available as supplementary data at *Bioinformatics* online and Section 3.1 for the full 149M-variant benchmark). GraphyloVar achieves an AUROC 0.674 in coding regions, 0.674 in cCREs, 0.652 in 3’ UTRs, and 0.655 in transposable elements, consistently outperforming all baselines. (B) Mean MAF (log scale) across variant score bins (1 to 20). GraphyloVar scores show clearer stratification of variants by frequency compared to competing methods, with higher-constraint bins corresponding to lower MAF, which is consistent with purifying selection.

Figure 2A shows the performance of GraphyloVar compared to established methods (GPN-MSA, GPN-Star, PhyloP, PhastCons, CADD, and Enformer) across different types of genomic contexts. For each method, we calculated the area under the Receiver Operating Characteristic curve (AUROC) to quantify how well each method distinguishes common from rare variants. The performance advantage was consistent across annotated genomic contexts (Fig. 2). Among UCSC-annotated variants, GraphyloVar achieves AUROC of 0.674 in coding regions, 0.674 in cCREs, 0.652 in 3’ UTRs, and 0.655 in transposable elements. Note that Fig. 2A shows only annotated variants; unannotated intergenic variants are excluded from this panel. On the full held-out set of ∼149M variants (including unannotated intergenic regions), the overall AUROC is 0.6246 (Fig. S2, available as supplementary data at *Bioinformatics* online); the lower value reflects the more difficult discrimination task when intergenic variants, which have weaker conservation signal, are included. Region-resolved values on the complete holdout set are provided in Table S1, available as supplementary data at *Bioinformatics* online.

The relationship between predicted scores and actual allele frequencies is further illustrated in Fig. 2B, which plots the mean minor allele frequency (log scale) across score bins (1–20) for each method. The ground truth panel (variants sorted by true MAF) shows the expected steep inverse relationship, with rare variants in the low-MAF bins. Among the prediction methods, GraphyloVar scores demonstrate the clearest stratification of variants by frequency. CADD and the Enformer-based DNN show relatively flat results, indicating limited ability to discriminate between variants under different levels of selective pressure. PhyloP and GPN-MSA show moderate correlations with allele frequency. These results indicate that GraphyloVar achieves strong discriminative performance across different genomic region types. This advantage stems from the model’s ability to leverage both sequence-specific features through transformer encoders and phylogenetic relationships through GCNs.

### 3.2 Correlation with observed minor allele frequencies

To further evaluate how well different methods capture the evolutionary constraints that shape allele frequency distributions, we calculated the Spearman correlation between predicted scores and observed minor allele frequencies on the held-out chromosomes 13 to 22 variant set. As shown in Table 1, GraphyloVar achieved the highest correlation (0.164), exceeding all baselines. This higher correlation indicates that GraphyloVar’s predictions better reflect the underlying selective pressures that determine the frequency of genetic variants in human populations.

**Table 1:** Spearman correlation between predicted variant scores and observed minor allele frequency (MAF) on the held-out chromosomes 13–22 TOPMed variant set (∼149M variants).^a^

Method	Spearman ρ
GraphyloVar	0.164
GPN-MSA	0.143
GPN-Star	0.139
Enformer	0.131
PhyloP	0.099
CADD	0.081
PhastCons	0.058

a Higher values indicate stronger agreement with population-level selective constraints.

GraphyloVar’s integration of phylogenetic structure yields a stronger Spearman correlation with observed minor allele frequency (ρ=0.164) than all baseline methods (ρ ranging from 0.058 to 0.143 across baselines, including site-level conservation metrics, integrative frameworks, and MSA-aware models). By capturing both sequence-specific features and evolutionary relationships, GraphyloVar better distinguishes variants likely to be tolerated from those likely to be deleterious and thus maintained at lower frequencies.

### 3.3 Predicting variant effects in massively parallel reporter assays

**Figure 3:**
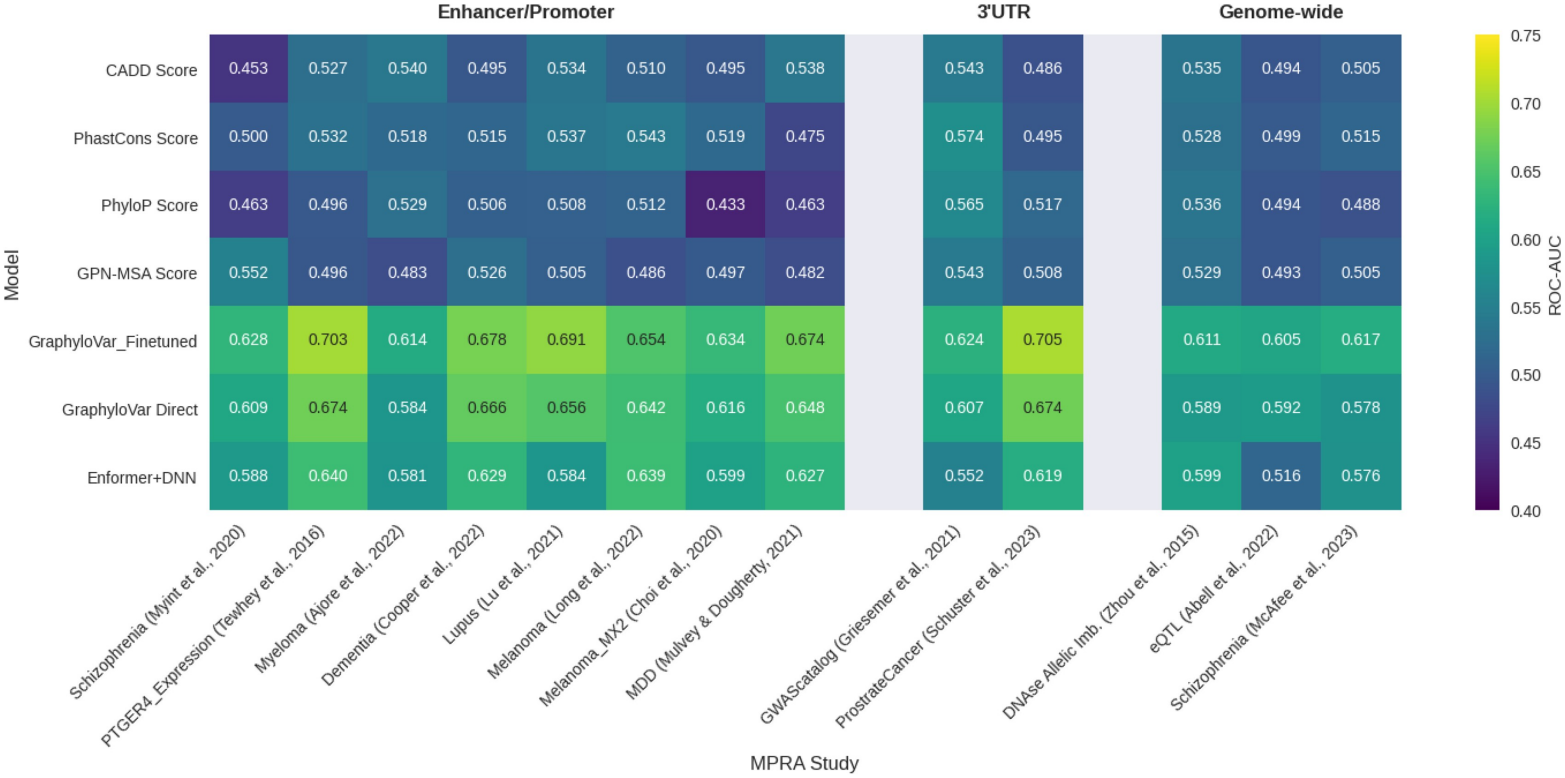
Heatmap showing AUROC values for different prediction methods (rows) evaluated on 13 MPRA datasets (columns) grouped by types of genomic contexts: enhancer/promoter regions (left), 3’ UTRs (middle), and genome-wide analyses (right). The bottom 3 rows show GraphyloVar fine-tuned on MPRA data (Graphylo_finetuned), GraphyloVar trained directly on MPRA labels without pre-training (Graphylo_Direct), and a DNN trained on Enformer features to predict MPRA positive SNPs; all three use leave-one-out cross-validation within each region group.

To evaluate GraphyloVar’s ability to predict functional effects of non-coding variants on gene regulation, we benchmarked our model against multiple MPRA datasets spanning diverse regulatory contexts. Figure 3 shows the performance (AUROC) of various prediction methods across 13 different MPRA studies organized into three types of genomic regulatory contexts: enhancer/promoter regions (8 datasets), 3’ UTRs (2 datasets), and genome-wide assessments (3 datasets).

#### 3.3.1 Performance improvements through fine-tuning

Given GraphyloVar’s ability to extract evolutionary signals, we explored two strategies to enhance performance: (i) fine-tuning the pre-trained model on MPRA data, and (ii) training a new GraphyloVar model directly on MPRA data without the evolutionary pre-training stage. Additionally, we compared these approaches with a fully connected deep neural network trained on Enformer features with MPRA binary labels (see Methods, Enformer paragraph).

GraphyloVar was fine-tuned separately for each type of genomic region and evaluated using a leave-one-out cross-validation approach to mitigate potential training biases. For example, for the enhancer/promoter group (8 MPRA datasets), we conducted eight independent experiments, where in each iteration, one MPRA dataset was designated as the test set while the model was fine-tuned on the remaining seven. This strategy ensures that the evaluation reflects the model’s ability to generalize across different datasets within the same regulatory region category. The same strategy was applied to the other two models.

As shown in Fig. 3, the fine-tuned GraphyloVar models (Graphylo_finetuned) demonstrated the strongest performance across all MPRA datasets, achieving the highest AUROC on all 13 datasets. In enhancer/promoter regions, this model achieved AUROC values ranging from 0.615 to 0.708. The GraphyloVar models trained directly on MPRA data without pre-training (Graphylo_Direct) and the DNN trained on Enformer scores also showed strong performance, but consistently below Graphylo_finetuned. These findings demonstrate that while GraphyloVar’s architecture captures core evolutionary signals that support zero-shot performance, incorporating task-specific experimental data through fine-tuning improves variant effect prediction. The pre-trained phylogenetic representations provide a generalizable foundation that the fine-tuning step adapts to MPRA-specific regulatory signals.

### 3.4 Complementarity of GraphyloVar scores with existing methods

**Figure 4:**
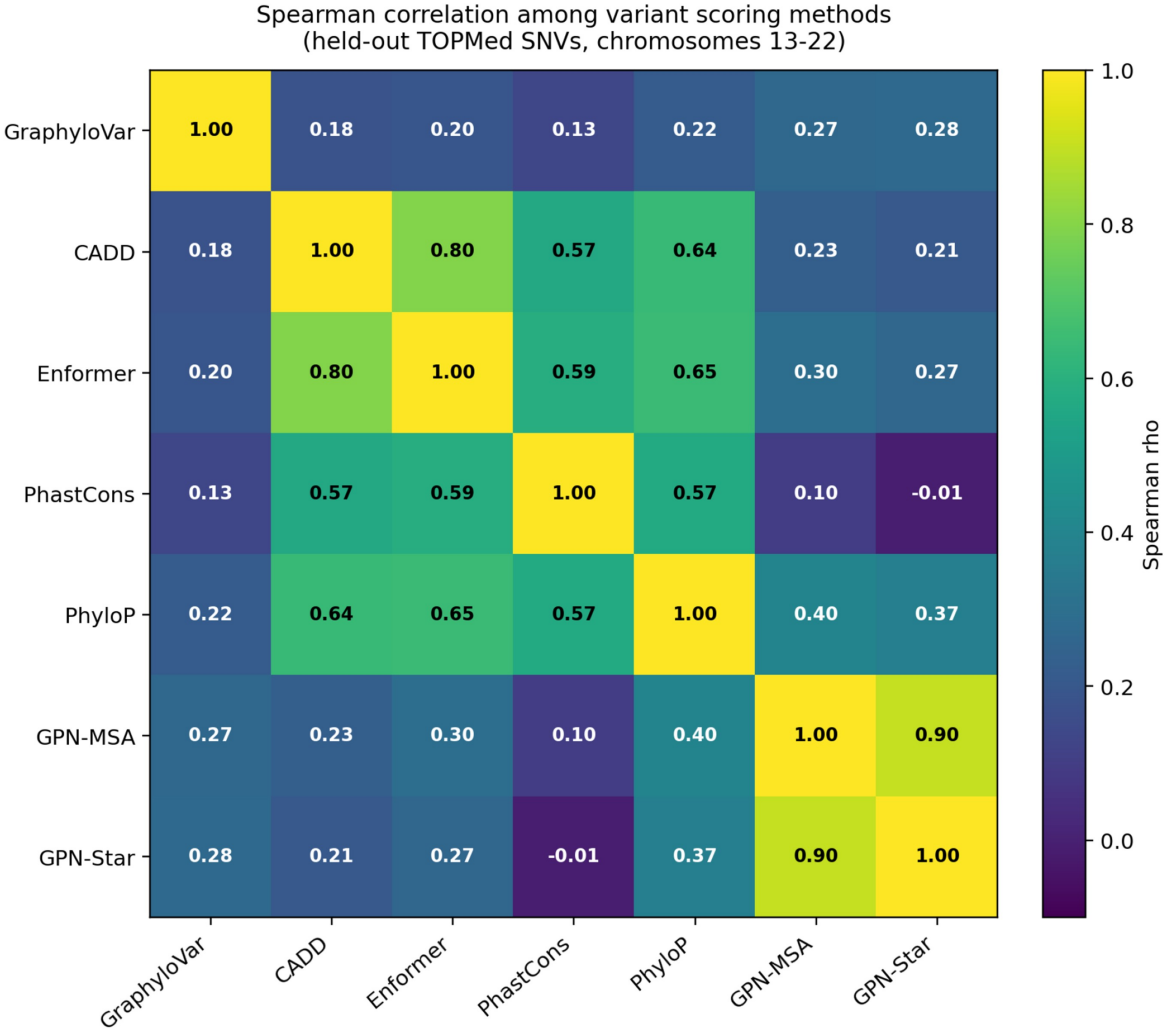
Spearman correlation among seven variant scoring methods (GraphyloVar, CADD, Enformer, PhastCons, PhyloP, GPN-MSA, GPN-Star), calculated on held-out TOPMed SNVs from chromosomes 13 to 22. GraphyloVar shows weak-to-moderate correlations with all baselines (ρ=0.13 to 0.28), indicating that it captures information distinct from both traditional conservation metrics and MSA-aware models. GPN-MSA and GPN-Star are highly correlated with each other (ρ=0.90), consistent with their shared architecture, yet both show only low correlation with GraphyloVar (ρ=0.27 and 0.28 respectively).

To assess whether GraphyloVar captures novel and complementary information compared to existing methods, we calculated the Spearman correlation between scores from different predictive approaches. Figure 4 presents a correlation matrix among genomic scoring methods, including GraphyloVar, PhastCons, PhyloP, CADD, Enformer, and GPN-MSA. Notably, GraphyloVar shows weak-to-moderate correlations with existing methods, indicating that it captures distinct information while still reflecting shared functional signals. As expected, traditional conservation metrics such as PhastCons and PhyloP show stronger correlations with each other than with GraphyloVar, which suggests that GraphyloVar captures signals beyond simple nucleotide-level conservation. We observe only weak correlation between GraphyloVar and major baselines such as CADD and PhyloP, indicating that the information captured by GraphyloVar is at least partially different from these existing methods.

To further investigate this, we combined GraphyloVar with CADD using a simple z-score ensemble: both scores are independently z-normalized (mean 0, standard deviation 1) over all ∼149M held-out variants. Because CADD is oriented so that higher scores indicate greater deleteriousness (i.e. rarer variants), its z-score is negated to align the direction with GraphyloVar. The ensemble score is the mean of the two sign-aligned z-scores. GraphyloVar achieves an overall held-out AUROC of 0.6246 on the full ∼149M TOPMed test set. Sign-aligned CADD achieves ≈0.5546. The z-score ensemble achieves AUROC of 0.6442, a gain of +0.020 over GraphyloVar alone (Fig. S3, available as supplementary data at *Bioinformatics* online; *P-*value$<10^{-15}$; paired two-sided DeLong test on 500 000 held-out variants). The low Spearman correlations together with the ensemble AUROC gain confirm that GraphyloVar captures information that CADD does not.

### 3.5 Context window ablation

To justify the default 65 bp context window (32 bp flank on each side of the variant, plus the variant position), we trained models with three context-window settings: flank ∈{16,32,100} base pairs, corresponding to input sequences of 33, 65, and 201 bp respectively. All models were trained on TOPMed chromosomes 1–10 with chromosomes 11–12 as validation, using the same training procedure (mixed precision, patience =30, Adam with ReduceLROnPlateau).

As shown in Table S1, available as supplementary data at *Bioinformatics* online, among the three context-window settings, flank =32 (65 bp) achieves the highest overall held-out AUROC (0.625), outperforming both the narrower flank =16 window (0.622) and the wider flank =100 window (0.617). The fine-tuned flank =32 model also achieves the highest AUROC across all 13 MPRA benchmark datasets (Section 3.3), confirming the 65 bp window as the best-performing setting across both zero-shot and fine-tuned evaluations. Exploring wider windows (flank >100 bp) may improve performance for tasks requiring longer-range regulatory context.

### 3.6 GCN interpretability and species importance

#### 3.6.1 Shape transition from mean pooling to GCN

In the original model, we summarized the species dimension using global average pooling, which collapsed the 115-species alignment into a single feature vector per position. This approach treats every species equally and discards the phylogenetic relationships among them.

In GraphyloVar, we replace mean pooling with a two-layer Graph Convolutional Network (GCN) that operates directly on the phylogenetic adjacency matrix. The input tensor has shape (B,115,F) where *B* is the batch size, 115 is the number of nodes (58 extant species + 57 inferred ancestors) in the placental mammalian evolutionary tree, and *F* is the feature dimension from the upstream Transformer encoder.

The first GCN layer transforms this to (B,115,32) by propagating features along edges of the phylogenetic graph, with each node aggregating information from its evolutionary neighbors weighted by the normalized adjacency matrix $\hat{A}=D^{-1/2}AD^{-1/2}$. The second GCN layer further refines these representations to (B,115,32). The output is then flattened to (B,3680) and passed through a shared fully connected layer (128 units, ReLU) before the dual-output prediction heads (allele frequency distribution and SNP probability), each with their own 64-unit dense layer.

This architectural change allows the model to learn species-specific weights that reflect evolutionary importance. An additive perturbation analysis (each species evaluated against an all-gap baseline on 500 000 held-out variants) shows that 53 of 58 extant placental mammals contribute positive isolated signal, with Human providing the largest contribution (ΔCE =7.1). Four diverged primates, Orangutan, Green monkey, Gorilla, and Gibbon, form a secondary cluster (ΔCE =0.62 to 1.50), and non-primate mammals contribute a broad gradient (ΔCE =0.002 to 0.12). Chimpanzee ranks 11th (ΔCE =0.062) despite being the closest relative of Human, because its ≈98.7% sequence identity with Human yields near-zero marginal (leave-one-out) contribution, reflecting information redundancy with the Human row (see Section 3.6). Five species (Baboon, Rhesus macaque, Marmoset, Squirrel monkey, Jerboa) show near-zero or marginally negative additive values, likely due to sparse alignment coverage at the tested variant sites. The GCN thus provides an interpretable mechanism for weighting conservation signals across the phylogenetic tree, rather than assuming uniform contribution from all species.

**Figure 5:**
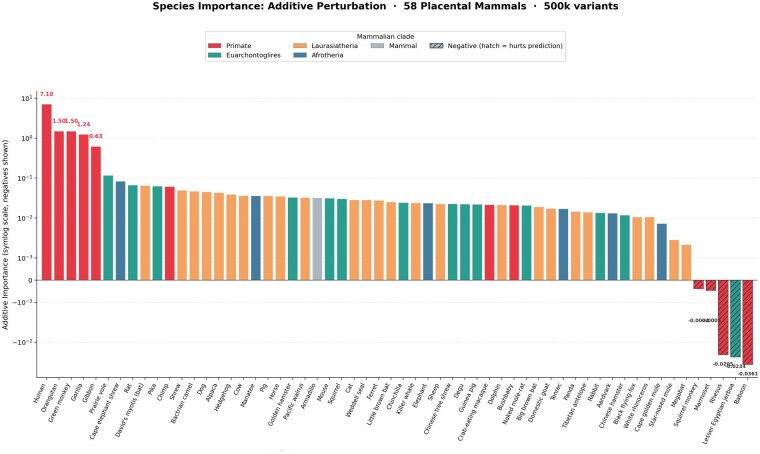
Additive species importance (Δ nucleotide cross-entropy when each species is added to an all-gap baseline) for all 58 extant placental mammals, evaluated on 500 000 held-out variants (chromosomes 13 to 22). Species are sorted by decreasing importance. The *y*-axis uses a symmetric log (symlog) scale with a linear region near zero, so that both positive and negative values are visible. Hatched bars indicate species whose isolated sequence slightly increases prediction loss relative to the all-gap baseline (i.e. negative additive importance): Squirrel monkey (−0.0004), Marmoset (−0.0005), Rhesus macaque (−0.0205), Jerboa (−0.0234), and Baboon (−0.0361); Human provides the dominant isolated signal (ΔCE =7.1). Among non-human species, four diverged primates, Orangutan (1.50), Green monkey (1.50), Gorilla (1.24), and Gibbon (0.63), form a clearly elevated cluster, followed by a broad band of non-primate mammals (0.002–0.12). Chimpanzee ranks 11th (ΔCE =0.062), consistent with information redundancy with the human row.

We investigated species-level importance using an additive perturbation analysis: for each of the 58 extant placental mammal species, we replaced all other alignment rows with a gap token and measured the decrease in nucleotide cross-entropy (Δ CE) relative to an all-gap baseline on 500 000 held-out variants (chromosomes 13 to 22). This quantifies the isolated information content of each species independently of the others. As shown in Fig. 5, 53 of 58 species provide positive additive signal, with Human supplying the largest contribution (ΔCE =7.1). Among non-human species, four primates form a prominent cluster: Orangutan (ΔCE =1.50), Green monkey (1.50), Gorilla (1.24), and Gibbon (0.62). Non-primate mammals show a broad range of smaller but positive contributions (ΔCE =0.002 to 0.12), demonstrating that the full multi-species alignment provides useful conservation signal beyond primates alone. Five species (Baboon, Rhesus macaque, Marmoset, Squirrel monkey, Jerboa) show near-zero or marginally negative additive values, likely because of sparse alignment coverage at the tested variant sites.

Chimpanzee, despite being the closest relative of humans, ranks 11th in additive importance (ΔCE =0.062), well below Orangutan (1.50). This is explained by information redundancy: Chimpanzee’s sequence is nearly identical to Human’s, so the model extracts similar evolutionary signal from both. The leave-one-out marginal contribution of Chimpanzee confirms this: masking Chimpanzee while Human is present yields ΔCE $\approx -2.4\times 10^{-6}$, which is within statistical noise, indicating that chimpanzee adds little incremental information on top of the Human row. In contrast, Orangutan, Green monkey, Gorilla, and Gibbon are sufficiently diverged to provide non-redundant context, explaining their higher importance in both additive and marginal analyses.

The SE-attention gate is also consistent with this pattern: the per-species sigmoid gate learned by the Squeeze-and-Excitation block assigns higher weights to species with more useful evolutionary signal. Human (≈1.00), Orangutan (0.54), Green monkey (0.40), Gibbon (0.34), and Gorilla (0.29) have the highest gate values, while Chimpanzee has the lowest gate among great apes (≈0.011), consistent with its near-zero perturbation importance. Species with near-zero or negative additive importance (Baboon $\approx 10^{-4}$, Marmoset $\approx 2\times 10^{-6}$) also have near-zero gate values. These results show that the learned attention weights match the perturbation importance rankings and that the model learns to weight species according to the phylogenetic tree structure rather than treating all species equally.

A phylogenetic tree ablation further confirms that performance increases monotonically as more species are included. Restricting the model to Human alone, then adding all Primates, and finally including the full placental mammalian tree each yields consistent AUROC gains across all genomic region categories (Fig. S2, available as supplementary data at *Bioinformatics* online), showing that non-primate mammalian sequences contribute useful evolutionary signal beyond the primate subtree.

### 3.7 Alignment coverage analysis

**Figure 6:**
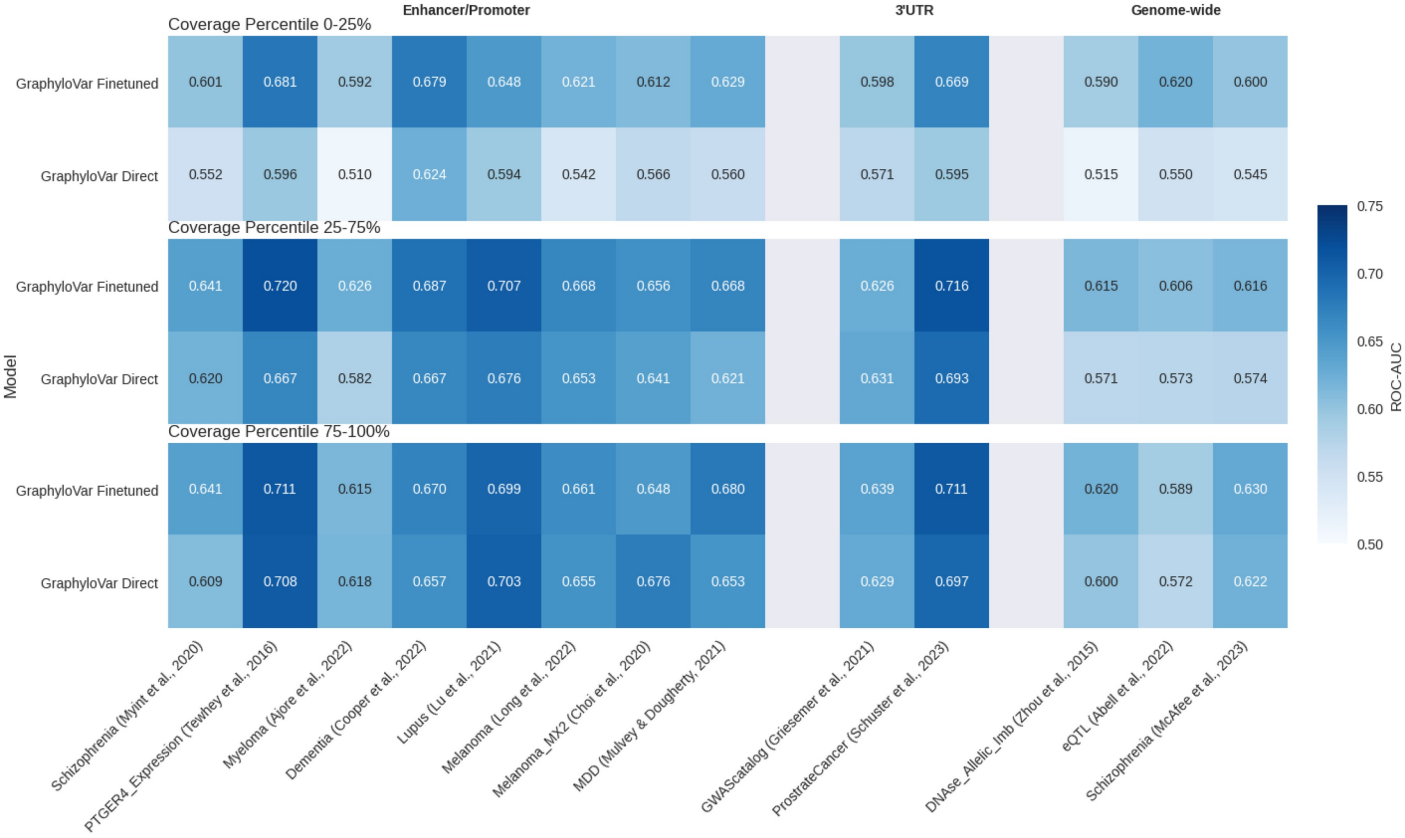
AUROC for fine-tuned GraphyloVar (Graphylo_finetuned) and directly trained GraphyloVar (Graphylo_Direct) models across three alignment coverage ranges (0–25%, 25–75%, 75–100%), evaluated on MPRA benchmark datasets. Alignment coverage is defined as the fraction of positions in the 65 bp window that contain observed nucleotides rather than gaps or missing data characters.

We investigated the impact of alignment coverage, defined as the fraction of positions in the 65 bp alignment window containing observed nucleotides rather than gaps (-) or missing data (N) characters. Figure 6 shows the performance of the Graphylo_finetuned and Graphylo_Direct models in three coverage percentile ranges: 0%–25% (low coverage), 25%–75% (moderate coverage), and 75%–100% (high coverage). The results reveal a clear relationship between alignment coverage and predictive accuracy in all genomic contexts. In the lowest coverage percentile (0% to 25%), both models show reduced performance. This is because sparse evolutionary information limits the Graph Convolutional Network’s ability to use phylogenetic relationships. Performance stabilizes between moderate (25% to 75%) and high (75% to 100%) coverage ranges, with similar AUROC values across both percentiles. These findings establish clear operating guidelines: regions with alignment coverage below 25% yield reduced predictive accuracy and should be interpreted with caution, while moderate-to-high coverage regions yield reliable predictions.

### 3.8 Computational time and scalability

While GraphyloVar’s incorporation of phylogenetic information enhances prediction accuracy, it introduces additional computational complexity compared to single-sequence models. GraphyloVar training required approximately 280 h on a single NVIDIA Quadro RTX 6000 GPU to complete pretraining on the TOPMed dataset. For prediction, GraphyloVar predicts approximately 20 variants per second on the same hardware.

## 4 Discussion and conclusion

GraphyloVar integrates deep learning and comparative genomics to predict the functional effects of genomic variants. By combining Transformer encoders for sequence feature extraction with Graph Convolutional Networks for phylogenetic modeling, the approach leverages multi-species alignments to identify evolutionary signals often missed by methods that focus on single-species sequences or site-level conservation metrics.

Our results demonstrate that GraphyloVar achieves competitive-to-superior performance across variant effect prediction benchmarks, with particular advantages in MAF stratification and fine-tuned MPRA prediction. The pre-trained model effectively distinguishes common from rare variants zero-shot, and after fine-tuning, accurately predicts regulatory effects measured in MPRA experiments. These advantages are especially apparent in non-coding regions, where traditional conservation-based approaches often struggle to identify functional elements subject to complex evolutionary constraints.

Pre-training on allele frequency prediction provides a biologically informed objective that captures evolutionary selective constraints more directly than conventional masked language modeling. By predicting population-level allele frequency distributions, the model internalizes the selective pressures shaping genomic variation, enabling effective zero-shot performance without requiring task-specific fine-tuning. GraphyloVar’s dual-output architecture, which predicts both allele frequencies and SNP probabilities, captures complementary aspects of evolutionary constraint. The primary score effectively identifies substitutions that replace evolutionarily favored nucleotides with disfavored ones, whereas the polymorphism score integrates information about the overall tolerance of variation at each position. This dual perspective aligns with the mechanistic understanding of how natural selection shapes genomic variation through both nucleotide-specific and position-specific constraints.

The interpretability analyses offer a partial mechanistic explanation for GraphyloVar’s performance. The additive species-importance analysis shows that 53 of 58 extant placental mammals contribute positive isolated signal, with Human providing the largest contribution (ΔCE =7.1) and four diverged primates (Orangutan, Green monkey, Gorilla, Gibbon; ΔCE =0.62 to 1.50) forming a distinct secondary cluster. Non-primate mammals contribute a broad but real gradient (ΔCE =0.002 to 0.12). Chimpanzee ranks 11th (ΔCE =0.062) despite its closest evolutionary proximity, because its ≈98.7% sequence identity with Human results in near-zero marginal contribution (leave-one-out ΔCE $\approx -2.4\times 10^{-6}$): the model has effectively learned that Chimpanzee’s signal is redundant with the Human row already in the alignment. These findings are consistent with a model that identifies nucleotide substitutions at constrained positions by integrating conservation signals across the full placental mammalian tree.

Despite these advantages, some limitations remain. The reliance on MSAs requires extensive computational time compared to single-sequence methods. Additionally, the current implementation focuses on substitutions rather than larger structural variants. A direct comparison with Evo2, a large-scale genomic foundation model, was not possible in this study because Evo2 requires GPU compute capability 8.9 or higher (Ada or Hopper architecture), while our hardware uses Quadro RTX 6000 GPUs (compute capability 7.5). We consider this an important future comparison. Future work could also address these constraints by exploring more efficient alignment processing, direct modeling of structural variants, integration with functional genomic data to capture tissue-specific effects, and wider context windows (flank >100 bp) that may better capture long-range regulatory interactions in enhancer and promoter contexts.

In conclusion, GraphyloVar introduces an approach for variant effect prediction that integrates deep learning with explicit modeling of phylogenetic relationships. The pre-training strategy, which predicts population-level allele frequencies, yields a foundation model that captures fundamental evolutionary constraints. Although the pre-trained foundation supports zero-shot variant prioritization, fine-tuning on task-specific experimental data (such as MPRA measurements) improves regulatory effect prediction. By leveraging evolutionary information through this pre-training and fine-tuning paradigm, GraphyloVar provides a complementary tool for prioritizing functionally relevant non-coding variants.

## Supplementary Material

btag426_Supplementary_Data

## Data Availability

GraphyloVar source code, trained model weights (flank = 32 checkpoint), and preprocessing scripts are available at https://github.com/DongjoonLim/GraphyloVar under the MIT License under DOI: 10.5281/zenodo.20616818. The TOPMed whole-genome sequencing data used for pre-training are available through dbGaP (accession phs000964). UCSC 100-way vertebrate whole-genome alignments are publicly available at https://hgdownload.soe.ucsc.edu/goldenPath/hg38/multiz100way/. MPRA datasets used for evaluation are available from the original publications cited in Section 2.5. Preprocessed data and relevant files can also be accessed at https://repo.cs.mcgill.ca/PUB/blanchem/GraphyloVar/.
