# Supplementary material for: GraphyloVar: predicting the impact of non-coding variants using a multi-species sequence model
Source: Bioinformatics. 2026 Jun 22;42(7):btag426. doi: 10.1093/bioinformatics/btag426 (PMC13330927; doi:10.1093/bioinformatics/btag426)
Supplement: btag426_Supplementary_Data [file btag426_supplementary_data.pdf]

Supplementary Material

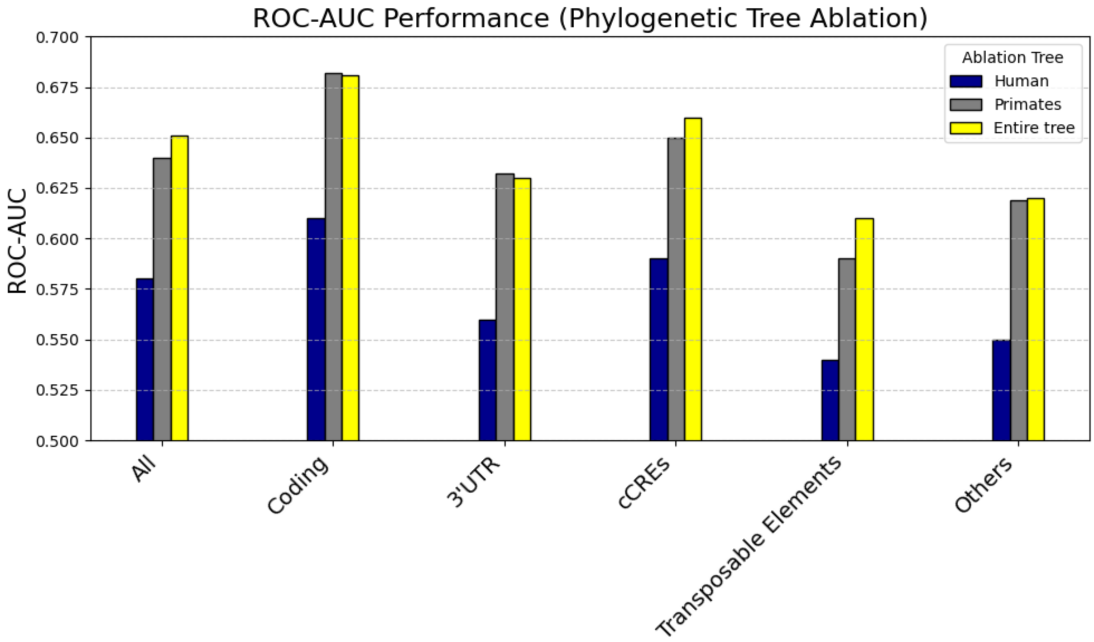

Fig. S1. Supplementary Figure S1: Phylogenetic tree ablation. Held-out AUROC when the model is restricted to the Human sequence only, to all Primate species, or to the full placental mammalian tree, evaluated across five genomic region categories (All, Coding, 3'UTR, cCREs, Transposable Elements). Performance increases monotonically as more of the tree is included, demonstrating that non-primate mammalian sequences contribute incremental but consistent signal beyond the primate subtree alone.

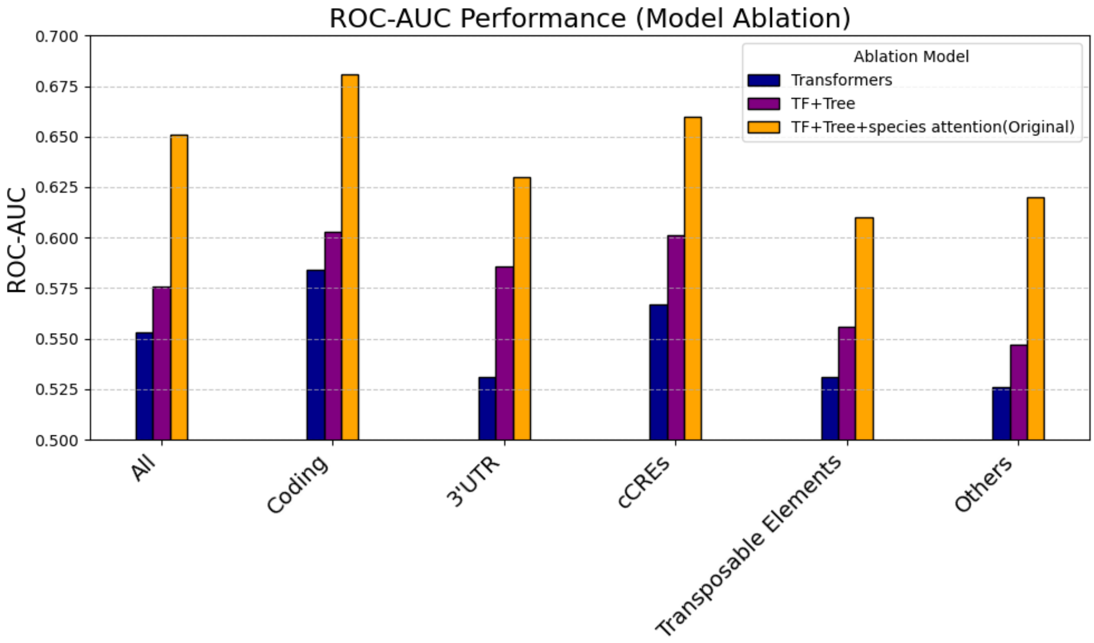

Fig. S2. Supplementary Figure S2: Model component ablation. Held-out AUROC for three model variants evaluated across six genomic region categories (All, Coding, 3'UTR, cCREs, Transposable Elements, and Other annotated regions). Transformers only: sequence encoder without any phylogenetic component. TF+Tree: Transformer encoder combined with the GCN phylogenetic tree, without the species-attention (SE) gate. TF+Tree+species attention (Original): the full GraphyloVar model. Each additional component yields a consistent AUROC improvement across all region types, confirming that the phylogenetic GCN is the largest single contributor beyond the sequence-only baseline, and that the SE attention gate provides further gains.

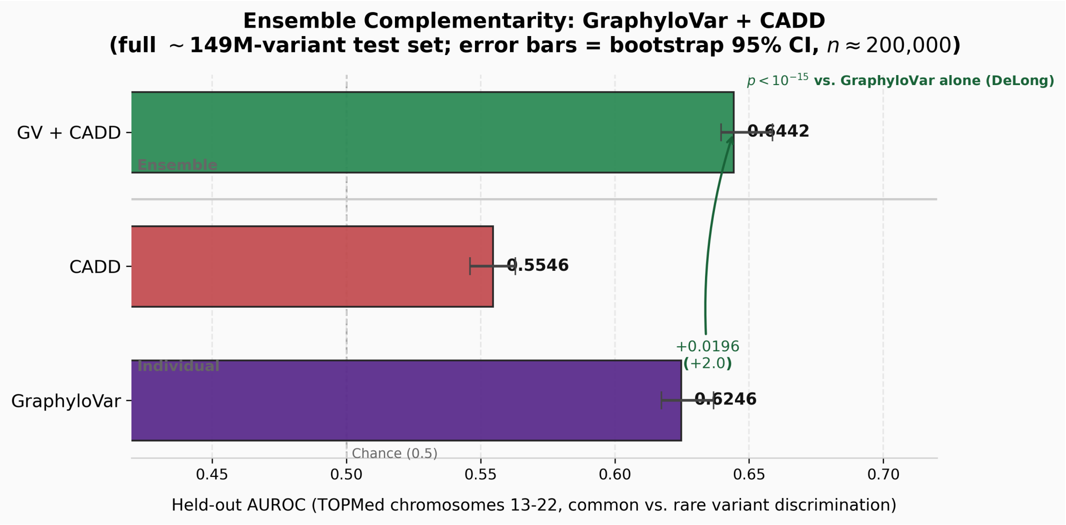

Fig. S3. Supplementary Figure S3: Ensemble. Held-out AUROC for individual variant scoring methods and the GraphyloVar + CADD z-score ensemble, evaluated on all ~149M TOPMed variants (chromosomes 13 to 22; common vs. rare variant discrimination). Unlike Figure 2A, which shows only UCSC-annotated region types, this benchmark includes the full variant set with unannotated intergenic sites, giving a baseline AUROC of 0.6246 for GraphyloVar. The ensemble is parameter-free: each score is independently z-normalized; CADD's z-score is negated to align polarity; the ensemble is the row-wise mean. Error bars show bootstrap 95% confidence intervals ($B = 1{,}000$, sub-sample $n \approx 200{,}000$). The ensemble achieves AUROC = 0.6442, a gain of +0.020 over GraphyloVar alone ($p < 10^{-15}$, DeLong test on 500,000 held-out variants). Non-overlapping bootstrap confidence intervals confirm that the ensemble gain is reliable and that GraphyloVar captures information that CADD does not.

Table S1. Supplementary Table S1: Context window ablation. Region-resolved held-out AUROC for three context-window sizes, evaluated on the full ∼149M TOPMed SNVs from chromosomes 13 to 22 (v3 architecture, embed_dim = 32, 2 attention layers). The main model (flank = 32, 65 bp) is indicated by †. Bold values indicate the highest AUROC per column.

Flank	Input (bp)	All	Coding	3' UTR	cCREs	TE
16	33	0.622	**0.625**	0.619	0.620	0.621
32†	65	**0.625**	0.616	**0.621**	**0.617**	**0.626**
100	201	0.617	0.610	0.616	0.617	0.616
